# Craniocervical Posture and Skeletal Malocclusion in Adolescents: A Cross-Sectional Study

**DOI:** 10.3390/jcm15051974

**Published:** 2026-03-04

**Authors:** Hande Ertem Arslan, Nuri Can Tanrısever, Mehmet Okan Akçam

**Affiliations:** Department of Orthodontics, School of Dentistry, University of Ankara, 06560 Ankara, Türkiye

**Keywords:** adolescent, cephalometry, malocclusion, posture

## Abstract

**Objective:** This study aimed to evaluate the relationship between craniocervical posture and skeletal malocclusion patterns in adolescents. **Methods:** This cross-sectional study included 80 adolescents aged 10–15 years diagnosed with skeletal Class I, Class II Division 1, Class II Division 2, or Class III malocclusion. Postural parameters—Sagittal Head Angle (SHA), Craniocervical Angle (CA), and Shoulder Angle (SA)—were assessed using standardized sagittal-plane digital photographs obtained in Natural Head Position. Skeletal classification and cephalometric measurements (SNA°, SNB°, ANB°, GoGn/SN°, and Occlusal Plane/SN°) were determined from lateral cephalometric radiographs. Intergroup comparisons were performed using the Kruskal–Wallis test, and posture–skeletal relationships were evaluated using Pearson and Spearman correlation analyses (*p* < 0.05). **Results:** No significant differences were observed in postural parameters among skeletal malocclusion classes (*p* > 0.05). In the overall sample, SHA showed weak negative correlations with SNA° (r = −0.284, *p* < 0.01) and SNB° (r = −0.381, *p* < 0.01), and a weak positive correlation with Occlusal Plane/SN° (r = 0.235, *p* < 0.05). No significant associations were identified for CA or SA. Subgroup analysis demonstrated that these associations were present exclusively in the Class II Division 2 group, where SHA showed strong negative correlations with both SNA° (r = −0.653, *p* < 0.01) and SNB° (r = −0.605, *p* < 0.01). **Conclusions:** Sagittal head posture may show phenotype-specific associations during adolescence, particularly in Class II Division 2 malocclusion. Incorporating postural assessment into orthodontic evaluation may enhance diagnostic understanding during growth.

## 1. Introduction

The craniofacial complex represents an integrated anatomical and functional unit composed of the craniomandibular and craniocervical systems. The craniomandibular system defines the functional relationship between the mandible and the cranium and plays a critical role in mandibular movements, mastication, phonation, temporomandibular joint function, and facial esthetics [1]. The craniocervical system consists of the cranium and upper cervical vertebrae and functions as a neuromuscular complex that regulates head posture, stability, and movement through coordinated muscle–ligament–bone interactions [2,3].

These systems are closely interconnected embryologically, anatomically, neuromuscularly, and functionally. Previous studies have described associations between mandibular morphology, sagittal jaw position, and head–neck posture, and cranio-cervical postural patterns have been reported in relation to craniofacial growth direction, including mandibular growth [4]. Conversely, changes in mandibular position—such as those observed after mandibular advancement—have been reported to coincide with modifications in natural head position [5]. These findings suggest a possible bidirectional association between the mandibular and cervical regions rather than a direct causal interaction. This relationship may be particularly relevant during growth and development.

Despite increasing interest in the relationship between posture and craniofacial morphology, evidence regarding the association between postural parameters and skeletal malocclusion patterns during adolescence remains limited and inconsistent, with previous studies reporting heterogeneous findings across different age groups and measurement protocols [6,7]. Recent systematic reviews have highlighted that, although associations between head and cervical posture and sagittal malocclusion are frequently reported, the overall certainty of evidence remains limited due to substantial heterogeneity in study design, age range, and outcome definitions. [7]. Furthermore, contemporary pediatric evidence examining the relationship between malocclusion and head, cervical, and whole-body posture similarly emphasizes variability across measurement protocols and malocclusion phenotypes [8].

Adolescence is characterized by significant craniofacial growth, particularly within the maxillomandibular complex [9,10]. Previous studies have reported associations between craniofacial morphology and craniocervical posture during growth [11,12]; however, the nature and direction of this relationship remain incompletely understood. More recent cross-sectional analyses stratified by growth periods have demonstrated that craniocervical posture parameters may vary across skeletal classes depending on maturational stage, suggesting that posture–skeletal associations may be growth-context dependent rather than universally present [13]. Consequently, focused investigations addressing postural–skeletal relationships in growing individuals are required.

Posture refers to the biomechanical alignment that determines the spatial orientation and functional relationship of body segments [14]. A physiologically balanced posture minimizes musculoskeletal stress, whereas deviations from normal spinal curvatures are associated with functional impairments and musculoskeletal disorders [15]. Physiological spinal curvatures develop around the age of seven, and postural abnormalities arising during childhood may either resolve during growth spurts or progress into structural deformities. The reported prevalence of postural abnormalities in children ranges from 33% to 41% between the ages of 7 and 15 years [16,17].

Postural deviations during growth may significantly influence craniofacial development. Björk et al. demonstrated that persistent posterior head positioning may contribute to posterior mandibular rotation and mandibular retrognathia through adaptive cranial base changes [18]. Solow et al. reported that increased soft-tissue tension associated with head extension redirects craniofacial growth vectors downward, resulting in increased anterior facial height, increased mandibular plane angle, and mandibular retrusion [19].

Conversely, craniofacial morphology itself may induce postural adaptations. Individuals with retrognathic facial morphology tend to maintain an extended head posture, whereas those with prognathic morphology often exhibit a flexed head posture [20]. In addition, sagittal discrepancies between the maxilla and mandible may displace the head beyond physiological postural limits. Associations have also been reported between cervical vertebral anomalies and skeletal malocclusions, particularly in Class III patterns, as well as between kyphotic posture and reduced SNB angles [21,22].

These findings support a relationship between head posture and craniofacial morphology; however, variability among malocclusion groups indicates that posture should be considered a contributing rather than a sole etiological factor [23]. Therefore, orthodontic evaluation should extend beyond dental and skeletal parameters to include neuromuscular and postural considerations.

Although posture–malocclusion relationships have been explored in previous studies, comparative analyses across clearly defined sagittal skeletal malocclusion subtypes during adolescence remain limited. In particular, few investigations have differentiated between Class II Division 1 and Class II Division 2 patterns when evaluating craniocervical posture, despite their distinct morphological characteristics. In line with this gap, recent reviews conclude that posture-related craniofacial associations may be pattern-dependent and should be interpreted with attention to subgroup characteristics rather than assuming uniform effects across sagittal classes [24]. Moreover, studies simultaneously examining posture parameters and cephalometric measurements within well-defined skeletal subgroups during active growth are scarce. Therefore, it remains unclear whether posture–skeletal associations differ according to specific sagittal malocclusion phenotypes in growing individuals.

The aim of this study was to evaluate the relationship between postural parameters and skeletal malocclusion patterns in adolescents and to determine whether specific skeletal patterns are associated with characteristic craniocervical postural features during growth.

The null hypothesis was that no significant association exists between postural parameters and skeletal malocclusion patterns or cephalometric characteristics in adolescents.

## 2. Materials and Methods

### 2.1. Study Design and Participants

This cross-sectional study included 80 adolescents who presented to the Department of Orthodontics, Ankara University Faculty of Dentistry, for orthodontic evaluation between February 2025 and July 2025. Participants were classified into four skeletal malocclusion groups: Skeletal Class I (mean age: 12.85 ± 1.57 years; 9 males, 11 females), Class II Division 1 (mean age: 12.21 ± 1.87 years; 12 males, 10 females), Class II Division 2 (mean age: 12.37 ± 1.57 years; 5 males, 14 females), and Class III (mean age: 11.68 ± 1.57 years; 8 males, 11 females).

Skeletal classification was performed using lateral cephalometric analysis according to Steiner analysis, based on ANB angle thresholds. Class II cases were further subdivided into Division 1 and Division 2 according to incisor inclination and overjet/overbite characteristics. Detailed classification criteria and cephalometric parameters are described in Section 2.5.

Individuals with no history of orthodontic or orthopedic treatment and without congenital anomalies or syndromes were included. Exclusion criteria comprised individuals who had completed their growth period, those presenting with temporomandibular joint dysfunction, and individuals with a history of cervical spine trauma, surgery, or diagnosed musculoskeletal disorders affecting head and neck posture, including clinically diagnosed spinal deformities such as kyphosis or scoliosis.

Completion of the growth period was determined using the cervical vertebral maturation (CVM) method as described by Baccetti et al. Lateral cephalometric radiographs were evaluated, and individuals classified as CVM stage 6 were considered to have completed active craniofacial growth and were therefore excluded from the study [25].

### 2.2. Ethics Approval and Consent to Participate

This study was conducted in accordance with the principles of the Declaration of Helsinki (World Medical Association, 2013) [26]. Ethical approval was obtained from the Ethics Committee of Ankara University Faculty of Dentistry (Approval date: 25 March 2024; Decision No: 36290600). Written informed consent was obtained from all participants and their parents or legal guardians prior to participation.

### 2.3. Postural Assessment and Natural Head Position Standardization

Postural assessment was performed using standardized lateral full-body photographs obtained in Natural Head Position (NHP).

NHP was first established clinically using a two-stage procedure combining neuromuscular self-balance and visual control. Participants were instructed to gently flex and extend their heads with progressively decreasing amplitude until reaching a comfortable and balanced position. They were then asked to look at their own eyes in a mirror positioned approximately 1.5 m away at eye level. The head posture obtained at this point was recorded as the Natural Head Position and used for photographic recording.

All participants were evaluated in a standardized standing position. Foot placement was controlled using predetermined floor markings to ensure consistent upright alignment prior to image acquisition. Photographs were obtained immediately after stabilization of the Natural Head Position.

A high-resolution digital camera (Canon EOS R6 Mark II, Canon Inc., Tokyo, Japan, 2022) was mounted on a tripod, positioned 115 cm above the ground and 150 cm from the participant, with the optical axis perpendicular to the sagittal plane to prevent parallax error. Anatomical landmarks were identified by palpation and marked with colored adhesive markers placed on the tragus, lateral canthus of the eye, spinous process of the seventh cervical vertebra (C7), and the acromion.

Standardized rectangular adhesive markers of identical dimensions were used for all participants. During digital analysis, the reference point was consistently defined as the midpoint of the marker (intersection of its horizontal and vertical axes) in ImageJ software. This approach minimized variability related to marker size and ensured consistent landmark identification. All landmark placements and angular measurements were performed by the same investigator under standardized conditions.

### 2.4. Postural Measurements

**Figure 1:**
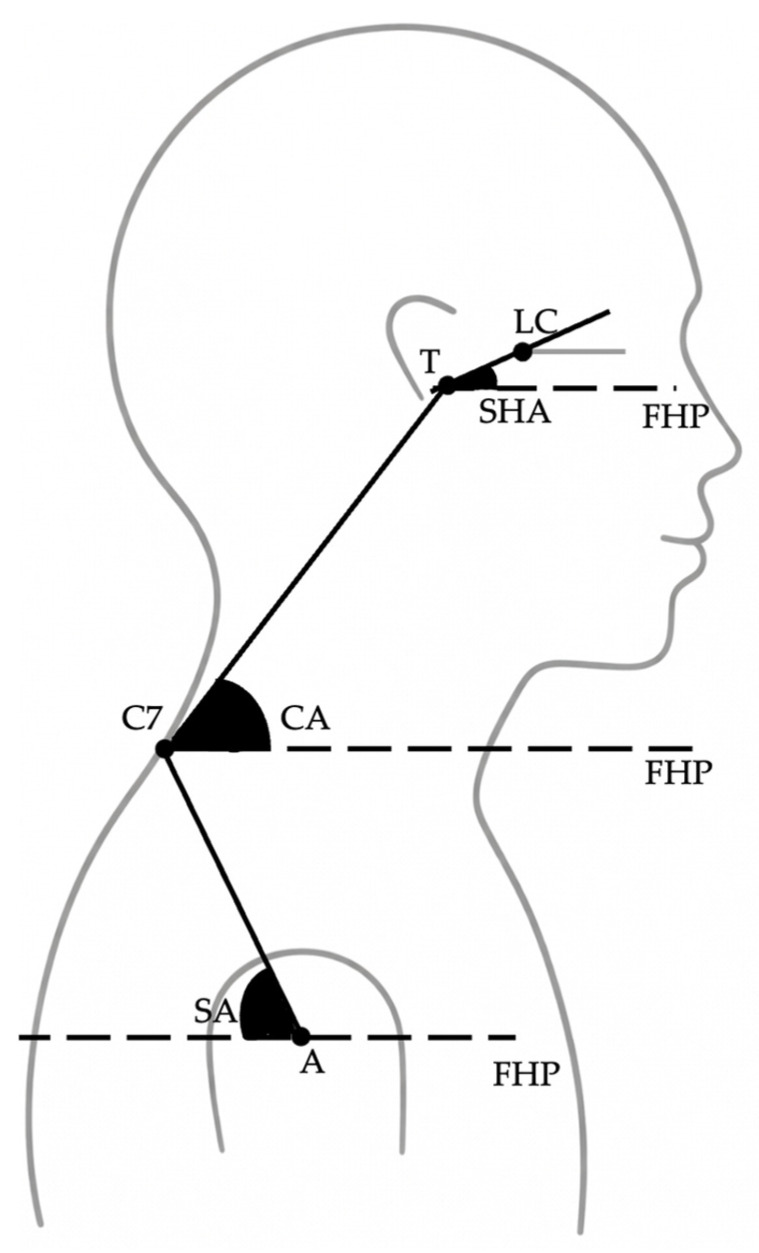
Anatomical landmarks and angular definitions used for postural assessment. The tragus (T), lateral canthus (LC), spinous process of the seventh cervical vertebra (C7), and acromion (A) were used to define the sagittal head angle (SHA), craniocervical angle (CA), and shoulder angle (SA). FHP represents the true horizontal reference line corresponding to the Frankfurt Horizontal Plane, used for all angular measurements.

**Figure 2:**
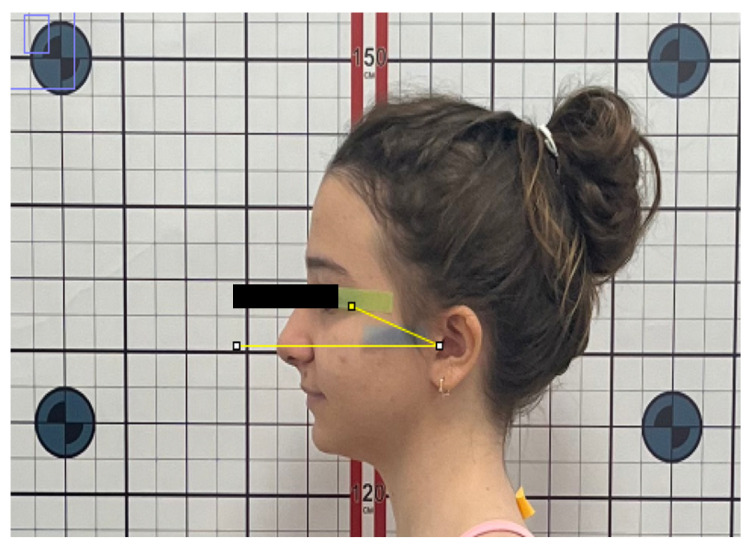
Sagittal Head Angle (SHA).

**Figure 3:**
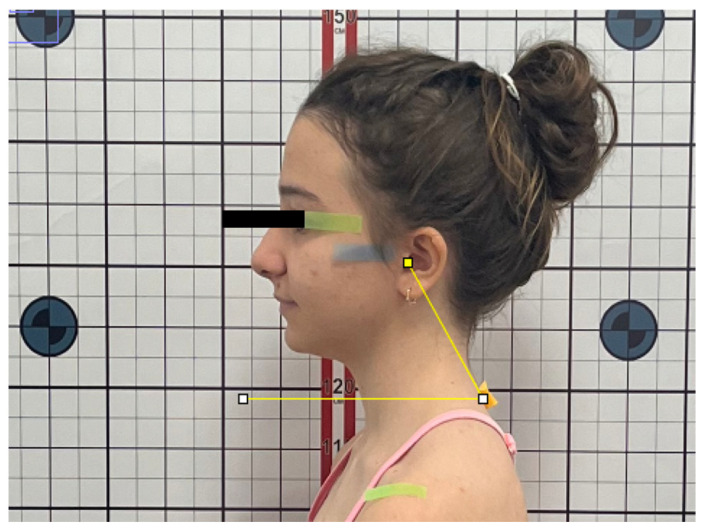
Craniocervical Angle (CA).

**Figure 4:**
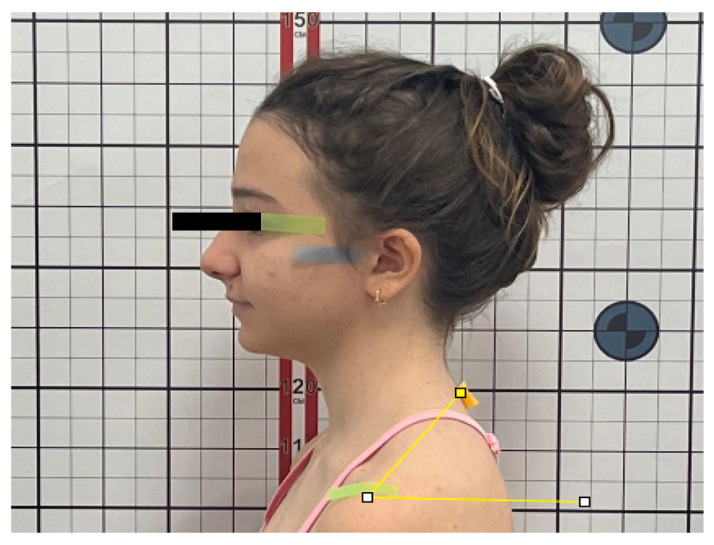
Shoulder Angle (SA).

To evaluate sagittal head and shoulder posture, the following angular measurements were recorded (Figure 1):

- Sagittal Head Angle (SHA): defined as the angle between the true horizontal reference line (FHP) and the line connecting the tragus to the lateral canthus of the eye (Figure 2).
- Craniocervical Angle (CA): defined as the angle between the true horizontal reference line (FHP) and the line connecting the tragus to the spinous process of the seventh cervical vertebra (C7) (Figure 3).
- Shoulder Angle (SA): defined as the angle between the true horizontal reference line (FHP) passing through the acromion and the line connecting the acromion to C7 (Figure 4).

All measurements were performed by a single investigator (H.E.A.) using ImageJ software (National Institutes of Health, Bethesda, MD, USA; version 1.54f, 2023).

### 2.5. Skeletal Classification

Skeletal classification and cephalometric measurements were performed on routinely obtained lateral cephalometric radiographs using Steiner analysis. The evaluated parameters included SNA°, SNB°, ANB°, GoGn/SN°, and Occlusal Plane/SN° angles. Skeletal patterns were defined as follows:

- Class I: ANB = 2° ± 2°;
- Class II: ANB > 4°;
- Class III: ANB < 0°.

Skeletal Class II cases were further categorized into Division 1 and Division 2 based on incisor relationship. Class II Division 1 was defined by increased overjet with proclination of maxillary incisors, while Class II Division 2 was characterized by deep overbite with retroclination of maxillary incisors.

In addition to their use for skeletal classification, SNA°, SNB°, ANB°, GoGn/SN°, and Occlusal Plane/SN° were also analyzed as continuous variables in correlation analyses to explore potential associations with postural parameters. These cephalometric measurements were not treated as primary intergroup comparative outcomes but were included to assess posture–skeletal relationships within the total sample and within each skeletal class.

To ensure that the same Natural Head Position established during photographic assessment was reproduced during cephalometric imaging, NHP was transferred to the cephalostat using the fluid-level (spirit level) method described by Showfety et al. [27].

### 2.6. Measurement Reliability

To assess intra-observer reliability, 20 randomly selected photographs were re-measured two weeks after the initial assessment by the same investigator. Intraclass correlation coefficients (ICC) were calculated using a two-way mixed-effects model with absolute agreement to evaluate the consistency of repeated angular measurements.

Repeated measurements included both landmark re-identification and angular recalculation, allowing indirect assessment of landmark placement consistency.

The investigator performing the postural and cephalometric measurements was not formally blinded to skeletal classification. However, all measurements were conducted using standardized digital protocols to minimize potential measurement variability.

### 2.7. Statistical Analysis

Statistical analyses were performed using SPSS software (IBM SPSS Statistics, version 26.0; IBM Corp., Armonk, NY, USA).

An a priori sample size calculation was performed using G*Power software (version 3.1; Heinrich-Heine-Universität Düsseldorf, Düsseldorf, Germany) for a one-way ANOVA (fixed effects, omnibus). Based on an assumed large effect size (f = 0.40), an alpha level of 0.05, and a desired statistical power of 80%, the required total sample size was calculated as 76 participants (19 per group). The final sample included 80 participants, exceeding the minimum required sample size and yielding an actual power of 0.82.

The normality of data distribution was assessed using skewness and kurtosis values, with values within ±2 considered indicative of normal distribution [28].

Intergroup comparisons of postural parameters among skeletal classes were performed using the Kruskal–Wallis test. When statistically significant differences were detected, pairwise comparisons were conducted using the Mann–Whitney U test.

The relationships between postural and cephalometric parameters were evaluated for the entire sample and within each skeletal class group. Pearson correlation analysis was applied for the overall sample, whereas Spearman’s rank correlation coefficient was used for subgroup analyses. Statistical significance was set at *p* < 0.05 (two-tailed).

## 3. Results

**Figure 5:**
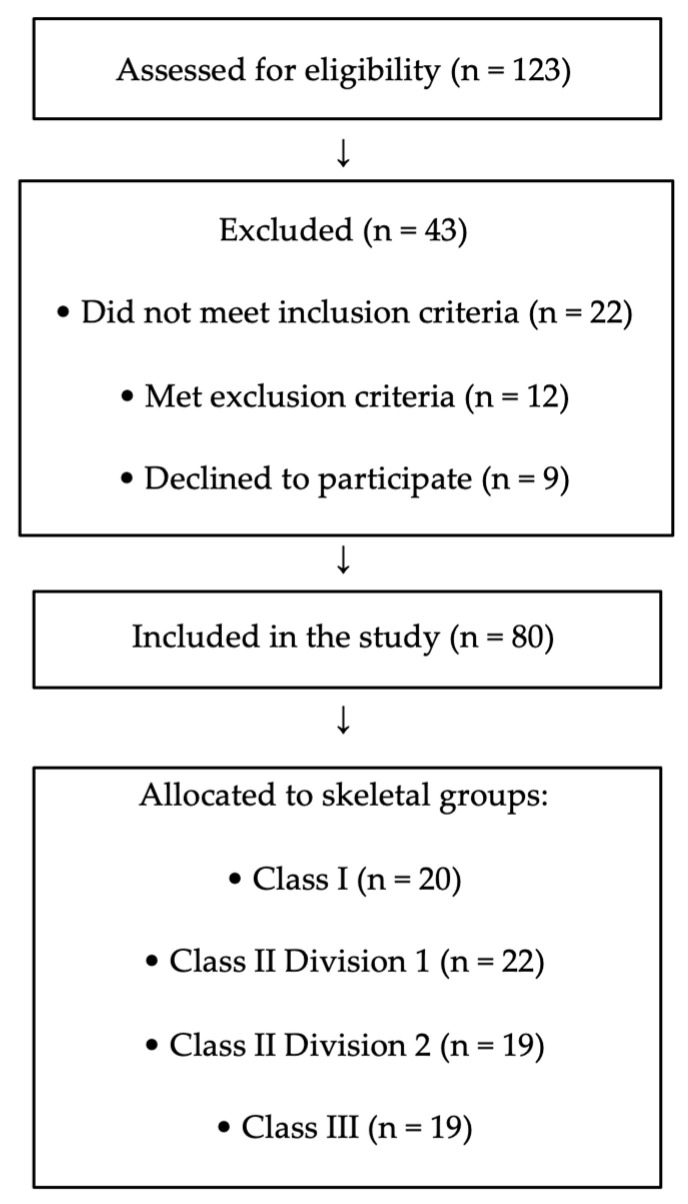
Flow diagram of participant recruitment and inclusion. A total of 123 adolescents were assessed for eligibility during the recruitment period. After applying inclusion and exclusion criteria and accounting for refusals, 80 participants were included in the final analysis and allocated to skeletal malocclusion groups.

During the recruitment period, 123 adolescents were assessed for eligibility. Of these, 22 did not meet the inclusion criteria, 12 met exclusion criteria, and 9 declined participation. The final sample consisted of 80 participants (Figure 5).

Intra-observer reliability analysis demonstrated excellent agreement for all angular measurements, with intraclass correlation coefficient (ICC) values exceeding 0.90.

Comparisons of postural parameters across skeletal malocclusion groups revealed no statistically significant differences in Sagittal Head Angle (SHA), Craniocervical Angle (CA), or Shoulder Angle (SA) (*p* > 0.05; Table 1).

Correlation analysis performed in the entire sample demonstrated that SHA was negatively correlated with SNA° (r = −0.284, *p* < 0.01) and SNB° (r = −0.381, *p* < 0.01), while a weak but statistically significant positive correlation was observed between SHA and Occ.Pl/SN (r = 0.235, *p* < 0.05). No significant correlations were identified between CA or SA and any cephalometric parameter (*p* > 0.05; Table 2).

When analyses were performed separately within skeletal malocclusion groups, no statistically significant correlations were observed in the Class I, Class II Division 1, or Class III groups (*p* > 0.05; Table 3, Table 4 and Table 5).

In contrast, within the Class II Division 2 group, SHA demonstrated statistically significant negative correlations with both SNA° (r = −0.653, *p* < 0.01) and SNB° (r = −0.605, *p* < 0.01), whereas no significant associations were found for CA or SA (Table 6).

## 4. Discussion

The present study investigated the relationship between craniocervical posture and skeletal malocclusion patterns during adolescence, a period characterized by active craniofacial growth and postural adaptation. Postural parameters were assessed using standardized photographic analysis in Natural Head Position, and their associations with cephalometric variables were examined.

No significant differences in postural parameters were observed among skeletal malocclusion classes. This finding suggests that sagittal malocclusion classification alone does not determine postural characteristics. Sagittal classification based on the ANB angle reflects the anteroposterior maxillomandibular relationship but does not fully capture other dimensions of craniofacial growth and functional adaptation that may influence postural characteristics [29,30].

In the overall sample, low-grade negative correlations were identified between the Sagittal Head Angle and both SNA° and SNB°. This association may indicate a compensatory tendency toward head extension in individuals with more posterior maxillary and mandibular positioning. This direction of association is consistent with recent evidence syntheses suggesting a tendency toward posture–sagittal morphology relationships, while also underlining that the magnitude and clinical interpretability remain uncertain because many studies are cross-sectional and vary in how posture is recorded and quantified [7]. A weak positive correlation between the Sagittal Head Angle and Occlusal Plane/SN further suggests coordinated adaptation within the head–jaw complex. In contrast, no significant associations were observed between cephalometric parameters and either the Craniocervical Angle or Shoulder Angle, indicating that sagittal head posture appears more closely related to sagittal skeletal positioning than to cervical or shoulder posture.

Although these overall correlations were statistically significant, their modest magnitude suggests limited predictive value at the individual level. Therefore, sagittal skeletal parameters alone may not be sufficient to anticipate postural characteristics in routine clinical settings.

When evaluated within malocclusion subtypes, no significant correlations were observed in the Class I, Class II Division 1, or Class III groups. However, a distinct finding emerged in the Class II Division 2 group, where strong negative correlations were identified between the Sagittal Head Angle and both SNA° and SNB°. This indicates that increased head extension in these individuals is closely associated with posterior positioning of the maxilla and mandible, suggesting a malocclusion-specific posture–craniofacial interaction.

Previous studies have similarly reported associations between head posture and Class II malocclusion. AlKofide and Alnamankani demonstrated closer relationships between craniocervical posture and Class II patterns than Class I [31]. Vukicevic and Petrovic reported increased head extension in Class II individuals [32], while Sandoval et al. described interactions between mandibular rotation, cervical curvature, and head posture in skeletal Class II cases [33]. The present findings extend this understanding by demonstrating that this relationship is particularly pronounced in Class II Division 2 malocclusion.

Importantly, most previous investigations have evaluated Class II malocclusion as a single entity. The present subgroup-specific analysis suggests that Division 2 morphology may represent a distinct functional and postural phenotype, thereby providing a possible explanation for inconsistencies reported in earlier literature.

The pronounced association observed specifically in the Class II Division 2 group may be related to the distinct morphological and functional characteristics of this malocclusion subtype. Class II Division 2 patients are typically characterized by retroclined maxillary incisors, deep overbite, and a tendency toward mandibular retrusion often accompanied by anterior mandibular rotation. This craniofacial configuration may be associated with increased elevator muscle activity and altered perioral muscle function, which can influence both mandibular positioning and head posture. From a biomechanical perspective, deep-bite morphology and posterior mandibular positioning may modify the functional balance of the suprahyoid and infrahyoid musculature, potentially affecting craniocervical alignment. Increased head extension in these individuals may therefore represent a compensatory neuromuscular adaptation aimed at maintaining functional equilibrium within the craniofacial–cervical complex. Such phenotype-specific adaptations may explain why posture–skeletal correlations were more evident in this subgroup than in other sagittal malocclusion patterns.

Posture–malocclusion relationships are known to be multifactorial and not limited to the sagittal dimension. Vertical growth patterns, airway considerations, and transverse skeletal asymmetries have all been implicated in postural adaptations [34,35,36,37,38,39]. However, in the present study, the vertical growth pattern assessed by GoGn/SN showed no significant association with postural parameters, supporting the variability reported in the literature.

From a clinical perspective, these findings suggest that postural evaluation may be particularly relevant in adolescents with Class II Division 2 malocclusion. Early identification of craniocervical extension patterns may assist clinicians in recognizing potential functional adaptations during growth. While orthodontic treatment alone is not expected to directly correct postural deviations, awareness of these associations may support more individualized treatment planning, particularly in deep-bite correction and growth-modification strategies. In selected cases, interdisciplinary collaboration—such as referral for physiotherapy-based cervical stabilization exercises or postural education—may be considered when clinically indicated. However, these findings should not be interpreted as an indication for routine postural intervention in all malocclusion patients.

This study has several limitations. An a priori sample size calculation was performed to ensure adequate statistical power for detecting clinically meaningful intergroup differences. Although the final sample met the required threshold, the study was primarily powered to detect relatively large effects. Consequently, more subtle posture–skeletal associations may not have been identified, and non-significant findings—particularly within subgroup analyses—should be interpreted with appropriate caution.

Additionally, the investigator performing the measurements was not formally blinded to skeletal group allocation, which may represent a potential source of measurement bias. However, standardized digital measurement protocols and excellent intra-observer reliability reduce the likelihood of systematic error.

Furthermore, postural assessment was limited to static sagittal-plane analysis, and malocclusions were primarily evaluated in the sagittal dimension. Future studies incorporating three-dimensional skeletal analysis, dynamic postural evaluation, and longitudinal designs may further clarify the complex interactions between posture and craniofacial development.

## 5. Conclusions

Postural parameters did not differ significantly among skeletal malocclusion classes in adolescents. However, a distinct posture–craniofacial relationship was identified in individuals with Class II Division 2 malocclusion, where increased head extension was closely associated with posterior maxillomandibular positioning.

These findings indicate that posture–malocclusion interactions are multifactorial and dependent on specific skeletal patterns rather than malocclusion classification alone. Incorporating postural assessment into orthodontic evaluation, particularly in Class II Division 2 patients, may contribute to more comprehensive diagnostic assessment and interdisciplinary management during growth.

## Figures and Tables

**Table 1:** Postural parameters across skeletal malocclusion groups (Kruskal–Wallis test).

Measurement	Class I Median (Min–Max)	Class II Div 1 Median (Min–Max)	Class II Div 2 Median (Min–Max)	Class III Median (Min–Max)	*p*
Sagittal Head Angle (°)	19.37 (7.91–34.13)	22.71 (11.37–30.67)	25.75 (9.34–35.70)	21.67 (12.53–31.76)	0.091
Craniocervical Angle (°)	52.36 (39.16–62.17)	52.94 (32.98–60.09)	48.45 (39.10–57.83)	50.79 (40.10–78.45)	0.248
Shoulder Angle (°)	56.29 (34.33–75.03)	56.58 (35.62–80.76)	56.57 (27.11–78.58)	58.70 (40.31–68.00)	0.944

Abbreviations: Min, minimum; Max, maximum. Significance level: *p* < 0.05.

**Table 2:** Correlation between postural and lateral cephalometric parameters in the total sample (Pearson correlation).

Cephalometric Parameter	SHA (r)	SHA (*p*)	CA (r)	CA (*p*)	SA (r)	SA (*p*)
SNA (°)	−0.284	0.0106 **	−0.007	0.950	−0.127	0.262
SNB (°)	−0.381	0.0005 **	0.048	0.675	−0.113	0.318
ANB (°)	0.155	0.170	−0.067	0.556	−0.001	0.993
Occlusal Plane/SN (°)	0.235	0.036 *	0.071	0.535	0.140	0.215
GoGn/SN (°)	0.166	0.142	−0.028	0.804	0.127	0.260

Abbreviations: SHA, Sagittal Head Angle; CA, Craniocervical Angle; SA, Shoulder Angle. r: Pearson correlation coefficient. * *p* < 0.05; ** *p* < 0.01.

**Table 3:** Correlation between postural and cephalometric parameters in the Class I group (Spearman correlation).

Cephalometric Parameter	SHA (r)	SHA (*p*)	CA (r)	CA (*p*)	SA (r)	SA (*p*)
SNA (°)	0.080	0.738	−0.099	0.679	−0.202	0.392
SNB (°)	0.129	0.588	0.008	0.973	−0.205	0.385
ANB (°)	−0.190	0.423	−0.272	0.247	0.002	0.993
Occlusal Plane/SN (°)	−0.108	0.652	−0.232	0.325	0.331	0.155
GoGn/SN (°)	0.173	0.467	0.084	0.724	−0.008	0.973

Abbreviations: SHA, Sagittal Head Angle; CA, Craniocervical Angle; SA, Shoulder Angle. r: Spearman’s rank correlation coefficient.

**Table 4:** Correlation between postural and cephalometric parameters in the Class II Division 1 group (Spearman correlation).

Cephalometric Parameter	SHA (r)	SHA (*p*)	CA (r)	CA (*p*)	SA (r)	SA (*p*)
SNA (°)	−0.177	0.456	0.382	0.096	−0.055	0.817
SNB (°)	−0.172	0.470	0.315	0.176	−0.057	0.810
ANB (°)	0.171	0.472	0.198	0.403	0.222	0.347
Occlusal Plane/SN (°)	0.016	0.946	−0.084	0.724	0.104	0.664
GoGn/SN (°)	−0.130	0.586	−0.085	0.722	0.220	0.351

Abbreviations: SHA, Sagittal Head Angle; CA, Craniocervical Angle; SA, Shoulder Angle. r: Spearman’s rank correlation coefficient.

**Table 5:** Correlation between postural and cephalometric parameters in the Class III group (Spearman correlation).

Cephalometric Parameter	SHA (r)	SHA (*p*)	CA (r)	CA (*p*)	SA (r)	SA (*p*)
SNA (°)	−0.358	0.122	−0.130	0.586	−0.249	0.289
SNB (°)	−0.344	0.139	−0.135	0.571	−0.296	0.205
ANB (°)	−0.012	0.960	−0.193	0.416	0.430	0.058
Occlusal Plane/SN (°)	0.256	0.276	0.089	0.711	0.000	1.000
GoGn/SN (°)	0.067	0.778	−0.211	0.372	0.102	0.666

Abbreviations: SHA, Sagittal Head Angle; CA, Craniocervical Angle; SA, Shoulder Angle. r: Spearman’s rank correlation coefficient.

**Table 6:** Correlation between postural and cephalometric parameters in the Class II Division 2 group (Spearman correlation).

Cephalometric Parameter	SHA (r)	SHA (*p*)	CA (r)	CA (*p*)	SA (r)	SA (*p*)
SNA (°)	−0.653	0.0018 **	−0.430	0.058	−0.265	0.260
SNB (°)	−0.605	0.0046 **	−0.309	0.186	−0.230	0.329
ANB (°)	−0.067	0.778	−0.153	0.522	−0.298	0.202
Occlusal Plane/SN (°)	0.330	0.156	0.044	0.853	0.033	0.890
GoGn/SN (°)	0.177	0.454	0.102	0.666	0.082	0.732

Abbreviations: SHA, Sagittal Head Angle; CA, Craniocervical Angle; SA, Shoulder Angle. r: Spearman’s rank correlation coefficient. ** *p* < 0.01.

## Data Availability

The data supporting the findings of this study are available from the corresponding author upon reasonable request. The data are not publicly available due to privacy and ethical restrictions.

## Abbreviations

ANB	Angle between points A, Nasion, and B
CA	Craniocervical Angle
C7	Spinous process of the seventh cervical vertebra
FHP	Frankfurt Horizontal Plane (true horizontal reference line)
GoGn/SN	Mandibular plane angle relative to the SN plane
ICC	Intraclass Correlation Coefficient
LC	Lateral canthus
NHP	Natural Head Position
Occ.Pl/SN	Occlusal plane angle relative to the SN plane
SA	Shoulder Angle
SHA	Sagittal Head Angle
SNA	Angle between Sella, Nasion, and Point A
SNB	Angle between Sella, Nasion, and Point B
SN	Sella–Nasion line
